# Decisions, decisions

**DOI:** 10.7554/eLife.32011

**Published:** 2017-09-29

**Authors:** Peter Rodgers

**Affiliations:** eLifeCambridgeUnited Kingdom

**Keywords:** peer review, scientific publishing, bias, funding agencies

## Abstract

Journals are exploring new approaches to peer review in order to reduce bias, increase transparency and respond to author preferences. Funders are also getting involved.

If you start reading about the subject of peer review, it won't be long before you encounter articles with titles like Can we trust peer review?, Is peer review just a crapshoot? and It's time to overhaul the secretive peer review process. Read some more and you will learn that despite its many shortcomings – it is slow, it is biased, and it lets flawed papers get published while rejecting work that goes on to win Nobel Prizes – the practice of having your work reviewed by your peers before it is published is still regarded as the 'gold standard' of scientific research. Carry on reading and you will discover that peer review as currently practiced is a relatively new phenomenon and that, ironically, there have been remarkably few peer-reviewed studies of peer review.

For many years peer review meant only one thing for many journals – single-blind peer review. In this approach an author submits a manuscript to a journal, an editor assigns it to anonymous referees for peer review and, if the referee reports are favourable, a revised version of the manuscript is accepted for publication. However, irrespective of whether the manuscript is ultimately accepted or rejected, the identities of the reviewers remain hidden from authors and readers, and their reports are only ever seen by the editor and authors. 'Single blind' means that the reviewers can see who the authors are, but the authors cannot see who the reviewers are.

In recent years, however, the picture has changed and journals have started to experiment with other forms of peer review – such as double-blind peer review, open or transparent peer review, interactive public peer review, results-free peer review and, brace yourself, post-publication author-led invited open peer review. At the same time there have been dramatic changes in other areas of scientific publishing such as the growth of open access, the rise of mega-journals, the increasing popularity of preprints in the life sciences and the involvement of funders in various aspects of scholarly communication (see Box 1).

Box 1. Funders get involved

Another emerging trend is the increasing involvement of funders in scientific publishing. In 2016 the Wellcome Trust (which also funds eLife) launched Wellcome Open Research on the F1000 platform, and the Gates Foundation and the UCL Great Ormond Street Institute of Child Health (which is part of University College London) have announced plans to launch similar titles – Gates Open Research and UCL Child Health Open Research – later this year.

The European Union has also announced that it is investigating the possibility of funding an open-access platform that will allow the rapid publication of both preprints and peer-reviewed articles for research funded by its Horizon 2020 programme (which has a budget of €80 billion). More details are expected later this year, but the EU has announced that the platform will "contain mechanisms for open/collaborate/public peer review".

Wellcome Open Research was set up for three main reasons says Robert Kiley, Head of Open Research at Wellcome: "to speed up the publication of research, to improve research reproducibility, and to encourage the sharing of all research outputs". The platform has published more than 100 articles since it was launched in late 2016, making it one of the most popular titles for Wellcome-funded authors. A survey of authors carried out in April found that publication speed and the ability to share a variety of research outputs were the primary reasons why authors published on the platform. Non-traditional article formats published by Wellcome Open Research include Software Tool Articles, Data Notes and Research Notes (which can include 'single-finding papers').

The survey also explored attitudes to open peer review and the ways referees were selected: when asked "what effect has authors choosing their own referees had on the peer review?", a third felt that this might result in the referees being less critical, 45% thought that there is no difference between authors and editors choosing referees, and 64% thought that the process allows authors to ensure that the referees have the right expertise. (Authors were able to agree with more than one statement in response to this question.) It is impossible to determine if authors selecting referees results in reviews being less critical, wrote Michael Markie of F1000 in a blog post at the time, "but it is worth noting that reviewers have been prepared to 'not approve' papers and that the reviews – all publicly available – are on occasions highly critical".

At present Wellcome Open Research only accepts submissions from researchers who are funded by the Wellcome Trust. However, the platform's website also states: "We hope that other funders will follow our lead and that, over time, funder-specific platforms will merge into a single international platform, open to all researchers."

Many of these developments are interlinked: a key feature of mega-journals, for example, is that peer reviewers are asked to not pass judgment on the potential importance or significance of a manuscript; rather, at PLOS ONE for example, their role is "to determine whether a paper is technically sound and worthy of inclusion in the published scientific record". Preprints, on the other hand, are not peer reviewed at all, although many go on to be reviewed and published by journals.

## At the double

The pressure to move away from single-blind peer review has been building for a number of years. In 2008 the Publishing Research Consortium conducted a survey in which more than 3000 academics were asked a series of questions about peer review. One of the clearest messages to emerge from the survey was a lack of support for single-blind peer review: 56% of respondents reported a preference for double-blind peer review, with just 25% preferring single-blind peer review, and only 13% preferring open peer review. "The overwhelmingly most popular reason for preferring double-blind review," the authors of the report explained, "was that it was seen as a more objective process, removing potential biases due for example to the author's institution, race or country, or personal biases."

The publisher Taylor & Francis also found a "strong preference for double blind review" when it surveyed more than 7000 authors, reviewers and editors in 2015. However, there was also relatively little appetite for radical change among the respondents: "Overall this study still found a fairly conservative researcher view, demonstrating a wish for tweaks to the current systems rather than a radically new way of assessing the quality and validity of research outputs."

Journals and publishers have been slow to react to these messages. However, at the recent Eighth International Congress on Peer Review, held September 10–12 in Chicago, two publishers – Springer Nature and IOP Publishing – reported the results of experiments in which authors were offered the option of single-blind or double-blind peer review for their manuscripts.

The Springer Nature trial involved over 100,000 submissions to Nature, Nature Communications and 23 other Nature journals (such as Nature Cell Biology) over a two-year period. Presenting the results in Chicago, Elisa De Ranieri of Springer Nature reported that 12% of authors had opted for double-blind review, and that there was no significant difference in the proportion of male and female corresponding authors selecting this option. This was surprising as it has long been thought that single-blind peer review was prone to gender bias. (About one-third of the manuscripts had to be excluded from this part of the analysis as the gender of the author could not be determined with sufficient confidence.)

The Springer Nature study also found that authors from the most prestigious institutions were the least likely to opt for double-blind review: only 4% of corresponding authors from institutions ranked in the top 10 of the 2016 Times Higher Education (THE) ranking opted for double-blind review, whereas the figure for institutions ranked 101 or below was 13%. And of the 10 countries that submitted the most manuscripts, authors from the US and China were the least and most likely, respectively, to select double-blind review.

De Ranieri and colleagues also studied whether the manuscripts in their sample were accepted or rejected. Nature journals use professional editors to make initial decisions on manuscripts: these editors typically reject about 80% of submissions without review, and send the remaining 20% to external referees. The Springer Nature study found that editors were less likely to send double-blind manuscripts to external referees (8% vs 23%), and that double-blind manuscripts were also less likely to be accepted after external peer review (25% vs 44%).

**Figure 1:**
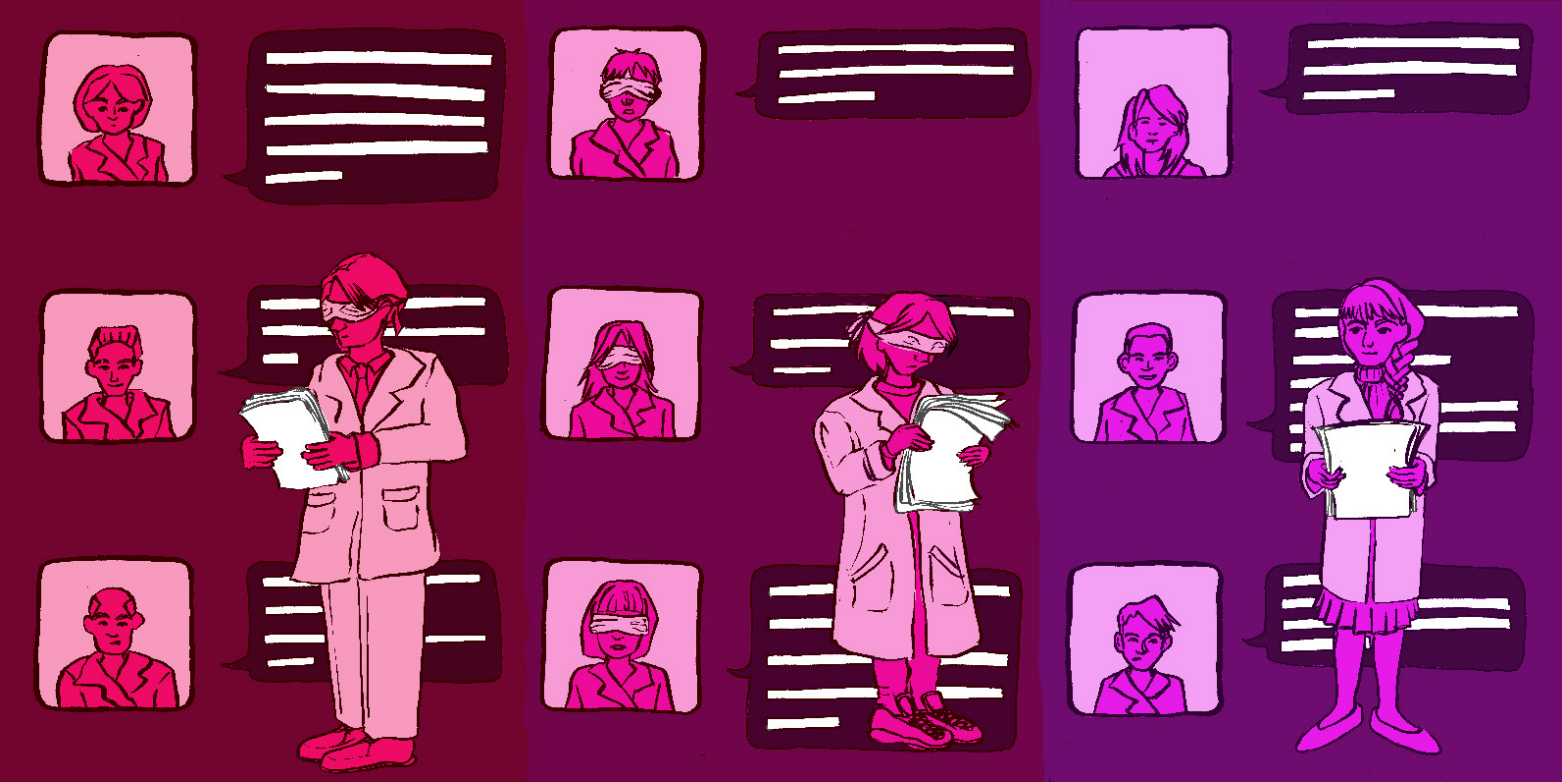
The many faces of peer review. While single-blind peer review is used by the vast majority of science journals, some offer double-blind or open peer review.

IMAGE CREDIT: vividbiology.com

The IOP Publishing pilot study offers the option of single- or double-blind review to authors submitting to two journals – Materials Research Express and Biomedical Physics & Engineering Express. Presenting preliminary results from the first seven months of the study (which will last a year), Simon Harris of IOP Publishing reported that 20% of authors had opted for double-blind review on both journals. Moreover, as with the Springer Nature study, the rejection rate for double-blind submissions was significantly higher than the rejection rate for single-blind submissions (roughly 70% vs 50%).

One of the next challenges, De Ranieri told the meeting in Chicago, is to determine if the different rejection rates are due to differences in quality or to bias on the part of editors and/or referees. To explore the possibility of bias by editors a number of Nature journals are thinking of experimenting with 'triple-blind review', which would involve professional editors making initial decisions on manuscripts without knowing who the authors were. It is also possible that the fact that an author has opted for double-blind review might bias referees against a manuscript: De Ranieri suggested that one way to overcome this form of bias, if it exists, would be to make double-blind review mandatory rather than optional. Another challenge for supporters of double-blind is that as preprints become more popular it will be increasingly difficult to stop referees knowing who the authors are.

## Open season

While single-blind and double-blind peer review are fairly well defined, the same is not true for open or 'transparent' peer review (see, for example, Tennant, 2017). In some journals it involves publishing the names of the referees with the paper, in others it involves publishing referee names and their reports, and in other journals it involves something else again. Indeed, in a recent review article in F1000Research, Tony Ross-Hellauer of Göttingen State and University Library reported how he had identified 22 distinct forms of 'open peer review' among scientific journals (Ross-Hellauer, 2017). Moreover, elements of open peer review can be optional: for example, some journals only publish the names of referees who have agreed to reveal their identity.

According to Ross-Hellauer, there are seven traits associated with open peer review, the most common trait being open identities (that is, publishing the names of referees), followed by open reports (publishing referee reports), and open participation (also known as crowd-sourced or public peer review because any researcher in a given field is able to review a manuscript). The other traits include open pre-review manuscripts (which includes preprint sites and titles like F1000Research, where manuscripts are available while they are being peer reviewed), and open interaction (which can involve the referees discussing their reports before a decision is reached). eLife employs a form of open interaction during which the editors and referees agree on the points that the authors need to address in order to have their paper accepted; the decision letter that contains this list of points is published with accepted papers, but the individual reports are not (King, 2017).

The BMJ is probably the highest-profile journal to employ open peer review and for each research article it publishes, it also publishes a 'prepublication history' that includes all previous versions of the article, signed referee reports, comments from BMJ editors and the authors' responses to these comments and reports. In 2011 the BMJ Group told a parliamentary committee looking into peer review that it had been using "signed open review [...] for more than a decade with no significant problems". However, it also noted that "*PLOS Medicine* tried and then discontinued this practice in late 2007 citing reviewers' reluctance to sign their reports".

The EMBO Journal also publishes a 'peer review process file' that includes referee reports and decision letters, among other things, but it is not compulsory for referees to reveal their identity. And Elsevier, one of the world's biggest scientific publishers, has plans to make open peer review an option on 1800 of its journals by 2020. "We've had positive feedback from our pilot titles," Philippe Terheggen, managing director of the company's journals group, told Research Information earlier this year, "so we now want to roll out open peer review across all Elsevier-owned journals."

"There are strong ethical arguments for open peer review," says Elizabeth Moylan, a senior editor at BioMed Central (BMC), which is part of Springer Nature. These include making editors and referees more accountable for the decisions made by journals, and providing credit for peer reviewers. Moylan adds that open peer review may help to train early-career researchers about the peer review process. More than 70 journals at BMC employ open peer review and publish named referee reports alongside papers.

So why is open peer review not more popular? One reason (apart from inertia and the lack of demand expressed in surveys; see above), is that journals find it more difficult to recruit reviewers. A recent study of how often researchers accepted invitations to review papers for a sample of BMC and SpringerOpen journals found that the accept rate was highest (60%) for double-blind journals and lowest (42%) for journals that used open review. The rate for single-blind journals in the sample was 53%. This echoes the findings of a study at the BMJ in the late 1990s which found that open peer review increased the likelihood of reviewers declining to review, but had "no important effect on the quality of the review, the recommendation regarding publication, or the time taken to review".

The biggest concern, however, is that reviewers may be reluctant to be sufficiently critical. As Jeffrey Flier, a former dean of Harvard Medical School, puts it in It's time to overhaul the secretive peer review process: "Some reviewers, especially junior scientists, fear that critical but honest reviews of work by senior scientists in the field would subject them to retribution by these senior figures, who might someday be asked to review their grants, papers, or promotions. Faced with such fears," Flier continues, "they might decline requests to do peer reviews, or provide less honest reviews seeking to pander to the authors. This isn't just a hypothetical concern – I have heard it from many faculty members at Harvard Medical School, as well as from some journal editors. To the extent it is real, this brings shame on a profession committed at its core to the pursuit of knowledge and truth."

In the same article Flier argues in favour of open peer review, with all referee names and reports being made public. "When peer review is cloaked in secrecy, there are limited incentives for performing high-quality reviews," he writes. "That allows bias, carelessness, conflict of interest, and other deficiencies to persist without a way to penalize those who generate inadequate reviews" (Flier, 2016).

## Publish first, review later

In 2001 the journal Atmospheric Chemistry and Physics introduced a twist to the peer review process called 'interactive public peer review'. In this approach a submitted manuscript is first subject to 'initial access peer review' to ensure that it falls within the scope of the journal and meets certain scientific and technical standards. The manuscript is then published as a 'discussion paper' to allow interactive discussion and public commenting by at least two designated referees (who can be anonymous or named) and other members of the scientific community. This 'open discussion' phase typically lasts eight weeks and the authors are expected to respond to comments as they appear. If all goes well the authors are asked to submit a revised manuscript, which the co-editor handling the paper can accept for publication in the journal or reject. (See the journal website for a full description of this process.)

When a paper is accepted, the previous versions, comments and author responses to comments remain on the site, although some referees opt to remain anonymous. In 2012 the journal's chief executive editor, Ulrich Pöschl of the Max Planck Institute for Chemistry, wrote that the journal's scientific reputation and low rejection rate (~15% at the time) "confirm that anticipation of public peer review and discussion deters authors from submitting low-quality manuscripts and, thus, relieves editors and referees from spending too much time on deficient submissions" (Pöschl, 2012).

F1000Research employs a similar form of peer review called 'post-publication author-led invited open peer review' and considers itself to be a 'platform' rather than a journal. Articles submitted to the platform are published as soon as they have passed certain basic checks (for example, the supporting data must be available), and then undergo post-publication peer review by referees who are suggested by the authors. "The editorial team checks the referee suggestions, and will only formally invite them to review the article if they fit our criteria – that is, if they are not close collaborators, have appropriate level of expertise, have no competing interests," says Sabina Alam, editorial director at F1000Research. "We believe that authors are best placed to identify appropriate experts for their paper, but we also believe it’s important that we check the suggestions carefully (including email addresses), and remain as the central point of contact between authors and referees, to ensure that our peer review standards are maintained."

At first articles are labelled as 'Awaiting peer review', with this label changing to a green tick (meaning a referee has 'approved' the manuscript), a green question mark (meaning an 'approved with reservations' rating) or a red 'x' (meaning a 'not approved' rating) when the first referee report is received. Further ratings are added as more reports are received, with the named referee reports being published shortly after they are received. Referee reports also have their own DOI, which means that they can be cited and included in the referee's ORCID profile: somewhat surprisingly, some journals that publish referee reports do not assign DOIs to them.

As with a traditional journal, authors can submit a revised version to address the points made by the referees and, they hope, convert question marks into ticks. An article is considered as accepted – and therefore suitable for indexing in PubMed and other bibliographic databases – when it receives two 'approved' ratings or one 'approved' and two 'approved with reservations' ratings. All versions of the article are available on the site, and when a new version is published, a summary is provided as to how that version differs from the previous version.

What advice does Alam have for any existing journal thinking of introducing an F1000-style peer review process? "The largest challenge is the technical infrastructure required to run the model," she says. "We had to build our own editorial management system and structure the underlying database in a very different way to most publishing systems, to enable us to incorporate the versioning and the referee reports being associated with specific versions. A skilled editorial team is also required."

A number of research funders have also started to use the F1000 platform for their own titles, or have plans to do so (see Box 1).

## Taking aim at publication bias

The tendency of authors and journals to publish positive results at the expense of negative or inconclusive results – a phenomenon known as publication bias – is also driving change in peer review. Two similar approaches, registered reports and results-free peer review, are trying to address this problem by focusing on the methods used in experiments rather than the results obtained.

In the registered reports approach the authors submit a 'stage 1 manuscript' that describes the hypotheses they plan to test and the methods they will use (Chambers, 2017). When this manuscript is peer reviewed, the referees focus on the hypotheses (are they well founded?) and the methods section (are the experimental designs and analysis procedures described in adequate detail? are the experiments well powered, do they contain adequate controls, and will they test the hypotheses?). If the reviews are positive the manuscript is accepted in principle, regardless of the results. A crucial feature is that data collection cannot start until the stage 1 manuscript has been accepted.

Once the experiments have been completed and the data analysed, the authors submit a 'stage 2 manuscript' which also includes a results section and a discussion section. If the authors have performed the experiments and analyses as described in the stage 1 manuscript, if any pre-specified quality checks (such as positive controls) have been passed, and if the conclusions of the study are justified by the data, the manuscript is accepted for publication.

The results-free approach to peer review has been available as an option to authors submitting to BMC Psychology since late 2016 (Button et al., 2016) and, as of mid-September 2017, the journal had published five papers that have been reviewed this way. In the results-free approach the author submits a partial manuscript that contains the rationale for the study and the methods section, but does not contain the results or the discussion. If the rationale and methods are deemed suitable and appropriate by the referees, the manuscript is accepted in principle, and the authors are asked to include their results and discussion, and to address any points raised by the referees, in a revised manuscript. Although the revised manuscript is re-reviewed, "the accept-in-principle decision can only be revoked at stage 2 if the results and discussion deviate unjustifiably from the stated aims and methods reviewed during stage 1" says Anna Clark, who was the editor of BMC Psychology until recently. The journal is also investigating the possibility of running a randomized control trial to determine if results-free peer review reduces publication bias compared with standard peer review (which is open peer review in the case of BMC Psychology).

To date over 60 papers have been published in 12 journals via the registered report route, with the majority appearing in psychology journals. Chris Chambers of Cardiff University – who is the editor for registered reports at Cortex, the European Journal of Neuroscience and Royal Society Open Science – estimates that another 100–200 have been provisionally accepted. All told, more than 60 journals now offer registered reports for regular issues, with others using the format for special issues. eLife, for example, employs a modified version of the format for the Reproducibility Project: Cancer Biology.

So what can be done to increase the use of this approach? "The most important practical step is for more journals to offer them," says Chambers. "All journals in all scientific fields that publish at least some articles reporting the outcomes of hypothesis testing should offer registered reports. This means we need thousands more journals to adopt the format." Chambers also argues that registered reports should be adopted in clinical trials to prevent publication bias and a practice known as 'outcome switching' (which involves authors not reporting outcome measures that were specified in the protocol for a clinical trial and/or reporting outcome measures that were not specified in the protocol). BMC Medicine recently became the first medical journal to offer a registered reports track for clinical trials.

And do registered reports have any disadvantages? "Perhaps the most common criticism is that they limit creativity or exploration in science, but this is not true," says Chambers. "Registered reports can, and often do, include extensive exploratory analyses, which are clearly labelled as exploratory so that readers can make informed judgments, but they are not designed to replace purely exploratory, non-hypothesis driven science." Cortex has plans to launch a new article type called Exploratory Reports for such research.

## Double act

In another development the journal Nicotine & Tobacco Research and the medical charity Cancer Research UK are collaborating on a pilot project that aligns decisions about funding and publication, with the journal agreeing, in principle, to publish the results of research that the funder decides to support. In the first stage of the 'registered reports funding' process a researcher submitting a standard application for funding to the charity's Tobacco Advisory Group can opt into the scheme: an editor from the journal who has been co-opted into the funding review process then checks that the work is within the scope of the journal, although the decision to fund remains solely with the funder. To begin just a small number of applications (randomly selected from those who have opted in) are being included in the pilot.

In the second stage the researcher submits a registered report about the work they have been funded to do, and the editor oversees the review of the report, using the same referees who reviewed the grant where possible. "Combining grant funding and publication decisions into a single, two-stage process promises to dramatically reduce the burden on reviewers, and also serve to reduce questionable research practices and publication bias," explained an editorial in the journal (Munafò, 2017). Nicotine & Tobacco Research is also collaborating on a similar project with a grants program called GRAND (short for Global Research Awards for Nicotine Dependence) that is funded by Pfizer.

So how will we know if the pilot has been successful? "Initially we just want to see what proportion of applicants are interested in this approach, and what their experiences of it are, and what proportion of grant reviewers agree to also review the registered report," says Marcus Munafò of Bristol University, who is the editor of Nicotine & Tobacco Research. "If the initial signs are positive we would like to run something similar again, and over time see whether it leads to improvements in the efficiency and quality of research published, if it protects against publication bias, and so on."

## Where next?

While many authors, reviewers and editors have different views on the best approach to peer review, they generally agree that scientific papers should be peer reviewed. However, a small minority disagree. Richard Smith, who was editor of the BMJ between 1991 and 2004, has long been a critic of peer review. In a highly-cited essay titled Peer review: a flawed process at the heart of science and journals, published in 2006, he wrote that peer review's "defects are easier to identify than its attributes. Yet it shows no sign of going away" (Smith, 2006). Ten years later he remains critical of the process – "Peer review is faith not evidence based, but most scientists believe in it as some people believe in the Loch Ness monster" according to a blogpost by Smith – but welcomes the approaches taken by Atmospheric Chemistry and Physics, F1000Research and Wellcome Open Research.

Not surprisingly there was little evidence of antithesis towards peer review in the programme for the recent congress in Chicago, although there were plenty of other things to worry about, such as conflicts of interest, various issues related to clinical trials and 'spin' (which has been defined as "reporting practices that distort the interpretation of results and mislead readers so that results are viewed in a more favourable light"; Chiu et al., 2017).

So, where next for peer review? In May this year Digital Science and BioMed Central released a report called What might peer review look like in 2030?. Based on a meeting held in London in late 2016, the report made seven recommendations for improving peer review: develop new ways (possibly based on artificial intelligence) to find suitable referees for papers; encourage more diversity in the reviewer pool; experiment with new models of peer review; invest in reviewer training programmes; encourage publishers to work together; develop ways to give reviewers credit for their work; and make greater use of technology (to, for example, develop automated ways to identify inconsistencies that are difficult for reviewers to spot).

Some of the recommendations are driven by the growth in the number of papers being submitted to journals, and the concomitant need to find more reviewers: increasing the numbers of referees from groups that are currently under-represented in the reviewer pool could help solve two problems at the same time. Concerns about the 'burden' of peer review could also be addressed if publishers worked together to make it easier for referee names and reports to be transferred between journals.

"Personally, I find it interesting that there is more research into peer-review itself, with interventions designed to improve the efficiency, transparency and accountability of peer review being more rigorously evaluated," says Anna Clark, who is now an assistant editor at BMJ Open. "Two good examples are the PEERE project, which is funded by the EU, and journals dedicated to peer review, such as Research Integrity and Peer Review."

## Final thoughts

It is common to read about actors, writers and directors who don't read reviews of their plays or films, and to hear novelists say that they don't pay attention to what the critics have said about their latest book. Scientists don't have this option: they have to read their reviews and to take on board what the reviewers have said, whether they agree with them or not. Nonetheless, if the system works as intended, the authors will benefit from the wisdom of their peers, readers will benefit from knowing that what they are reading has been subject to a form of quality control, and some small corner of science will take a small step forward. However, even when working as intended, it is clear that the peer review system remains prone to a range of biases, with particular fields wrestling with specific challenges, such as publication bias in psychology and outcome switching in the reporting of clinical trials. It is unlikely that we have seen the end of articles with titles like Can we trust peer review?, but the domination of single-blind peer review is certainly under assault from a range of alternatives.

## Note

This Feature Article is part of a collection of articles on peer review.
